# Predictors of seasonal influenza and COVID-19 vaccination coverage among adults in Tennessee during the COVID-19 pandemic

**DOI:** 10.3389/fpubh.2024.1321173

**Published:** 2024-03-04

**Authors:** J. Cunningham-Erves, W. George, M. Sanderson, E. Stewart, S. W. Jin, J. Davis, H. M. Brandt

**Affiliations:** ^1^Department of Internal Medicine, School of Medicine, Meharry Medical College, Nashville, TN, United States; ^2^Department of Health Policy, Vanderbilt University Medical Center, Nashville, TN, United States; ^3^Department of Family and Community Medicine, School of Medicine, Meharry Medical College, Nashville, TN, United States; ^4^School of Social Work, The University of Memphis, Memphis, TN, United States; ^5^Department of Biochemistry and Cancer Biology, School of Medicine, Meharry Medical College, Nashville, TN, United States; ^6^St. Jude Children’s Research Hospital, Department of Epidemiology and Cancer Control, Memphis, TN, United States

**Keywords:** vaccine uptake, COVID-19, influenza, flu, SARS-CoV-2, coronavirus

## Abstract

**Background:**

The COVID-19 pandemic has convoluted hesitancy toward vaccines, including the seasonal influenza (flu) vaccine. Because of COVID-19, the flu season has become more complicated; therefore, it is important to understand all the factors influencing the uptake of these vaccines to inform intervention targets. This article assesses factors related to the uptake of influenza and COVID-19 vaccines among adults in Tennessee.

**Methods:**

A cross-sectional, secondary data analysis of 1,400 adults was conducted in Tennessee. The adult sample came from two data sources: Data source 1 completed a baseline survey from January to March 2022, and data source 2 was completed from May to August 2022. Data on vaccine attitudes, facilitators and barriers, and communication needs were collected via random digit dial by Scientific Telephone Samples (STS). Two multivariable logistic regression models were used to estimate adjusted odds ratios (aORs) and 95% confidence intervals (CIs) to predict sociodemographic and overall vaccine-related factors associated with receipt or non-receipt (referent) of COVID-19 and influenza vaccines.

**Results:**

Approximately 78% of the adult sample had received the COVID-19 vaccination. A significant positive association for COVID-19 vaccine uptake was seen among those who were older (aged 50–65) (aOR = 1.9; 95% CI: 1.2–3.2), Black (aOR = 2.0; 95% CI:1.3–2.8), and had a college education and higher (aOR = 2.3; 95% CI: 1.5–3.6). However, there was a significant negative association for persons reporting they were extremely religious (aOR = 0.5; 95% CI:0.3–0.9). Over 56% of the adult sample had received the influenza vaccination this season. Those who had a higher annual household income ($80,000+) (aOR = 1.9; 95% CI: 1.3–2.6) and had health insurance (aOR = 2.6; 95% CI: 1.4–4.8) had a significant positive association with influenza vaccine receipt. However, those who were employed part-time or were unemployed had a significant negative association for influenza vaccine receipt (aOR = 0.7; 95% CI: 0.5–0.9). Both COVID-19 and influenza vaccine receipt had strongly significant positive trends with increasing belief in effectiveness and trust (*p* < 0.0001) and strongly significant negative trends with higher levels of overall vaccine hesitancy (*p* < 0.0001).

**Conclusion:**

Strategies to increase COVID-19 and influenza vaccination should be age-specific, focus on increasing geographical and financial access, and offer tailored messages to address concerns about these vaccines.

## 1 Introduction

Addressing vaccine hesitancy is a public health priority. It is a top threat to public health globally and in the United States (US). Hesitancy for vaccines remains a prominent reason for the delay or refusal of routinely recommended vaccinations, even though they are available (1–6). As a result, many individuals are left susceptible to harmful, infectious diseases that vaccination programs have eliminated or significantly reduced (7). Vaccine hesitancy occurs among adults for routinely recommended vaccines, as well as parents and caregivers making decisions for children regarding routinely recommended vaccines (8–10). In addition, studies suggest that vaccine hesitancy for one vaccine has fueled hesitancy for other vaccines, especially those that are not mandated (9). In the US, influenza vaccination uptake among adults usually hovers at approximately 50% (11). The same reticence exists among parents. Recent national data show that vaccination coverage for influenza remains suboptimal in children, with rates of 45.6% in December 2022 (12). In both cases, vaccine hesitancy has been cited as a major barrier.

The COVID-19 pandemic has fueled increased levels of hesitancy for vaccines, including seasonal influenza (flu) vaccines (13). COVID-19 vaccination uptake among adults has stalled at 69.5% fully vaccinated and 81.4% with one dose. Population-based studies further demonstrate that approximately 30% of the US population is hesitant to receive COVID-19 vaccines (14–17). Particularly, the Mid-South and Southeastern US have high rates of hesitancy for influenza and COVID-19 vaccines based on national surveys (18–22). Vaccine hesitancy is context and time-specific, varying among individuals (23). The National Adult Immunization Plan calls for the demand for adult immunizations to be increased in the community, which requires a vigilant, multi-pronged approach (24). At the root of these efforts, one must understand the factors influencing vaccine hesitancy for these strategies to increase vaccine acceptability while lowering hesitancy.

Determinants of vaccine hesitancy have been widely studied, with communication (e.g., social media and the Internet) being a major influencer of these factors. Common reasons for vaccine hesitancy include vaccine and vaccination-specific concerns (e.g., safety and side effects), contextual issues (e.g., politics, past research abuses, and access), and individual/group influences (e.g., lack of knowledge and misinformation) (23, 25). For example, Gallant et al. found that vaccination adherence in adults is higher when workplace vaccination campaigns are available, especially when coupled with information strategies addressing reasons for vaccine hesitancy (26). Yet, to the best of our knowledge, a handful of studies have determined if factors influencing vaccine hesitancy differ by vaccine type (8, 9). This warrants concern as people who are classified as vaccine-hesitant may have different or similar motivations and informational needs across vaccines (23) which could be overlooked in intervention development, limiting effectiveness. Furthermore, vaccine concerns change over time and should be monitored for changes in vaccination attitudes and behaviors for different vaccines (27).

The purpose of this study is to assess factors related to the uptake of influenza and COVID-19 vaccines among adults in Tennessee. This study’s novelty lies in being the first to explore influenza and COVID-19 vaccine concerns in Tennessee. Findings will be used to develop an intervention to be assessed in a future pilot efficacy study. Moreover, this study could inform intervention targets for future research to address adult vaccine hesitancy by vaccine type.

## 2 Participants and methods

### 2.1 Study design and data source

This cross-sectional study used secondary data from 1,400 adults who reside in Tennessee. Two data sources or survey samples were combined to create the study sample. Adults in data source 1 completed a baseline survey that was collected from January to March 2022, while survey completion for data source 2 was from May to August 2022. Both of these surveys were part of a pilot exploratory study to understand why vaccine-hesitant adults (data source 1) or vaccine-hesitant adults who are parents (data source 2) delay or refuse non-mandated vaccines (i.e., COVID-19, influenza, and human papillomavirus vaccines). Because no previous data were available during the time of the study, the sample size was based on our prior experience with the intent to use the data to calculate the sample size for a larger study. The 15-min surveys collected data on vaccine attitudes, facilitators and barriers, and communication needs among individuals. The likelihood of overlap between the survey study samples was small. Nonetheless, we created a unique identifier by concatenating 12 demographic variables to ensure the independence of the two survey samples.

Data source 1 was a survey with adults with the following inclusion criteria: (1) agreed to participate in the study, (2) between ages 18 and 65 years, (3) live in Tennessee, and (4) lived in the Nashville metropolitan statistical area (MSA), Memphis MSA, or Chattanooga MSA. This data source had 600 persons comprised of 100 African Americans per MSA (*n* = 300) and 100 individuals of all other ethnicities per MSA (*n* = 300). These adults are part of a larger study designed to compare attitudes and vaccination rates pre-post a social marketing campaign to increase COVID-19 vaccination in the Nashville MSA, with the Memphis and Chattanooga MSAs serving as the control.

Data source 2 was a survey of adults who were parents (i.e., parent survey) with the following inclusion criteria: (1) agreed to participate in the study, (2) lived in Tennessee, (3) lived in Nashville MSA (until 300 completed responses) or elsewhere in the state, and (4) a parent/primary caregiver of a child between the ages of 5–17 years. There was no age requirement for this data; however, nearly all participants were between ages 18 and 65 years (9 parents over age 65 were merged with the 50–64 age category). This data source was completed with 800 persons regardless of race, 300 adults from the Nashville MSA, and 500 adults from the rest of Tennessee. This study was approved by the Institutional Review Board of Meharry Medical College (Protocol #: 21-03-1076).

### 2.2 Study population

While Tennessee comprises 6.8 million people, the MSAs in our study include 21% of the population. The Nashville MSA has 14 counties (i.e., Davidson, Cannon, Williamson, Wilson, Rutherford, Cheatham, Hickman, Macon, Maury, Robertson, Smith, Sumner, Trousdale, and Dickson). The Memphis MSA comprises three counties (i.e., Shelby, Tipton, and Fayette). The MSA of Chattanooga has three counties (i.e., Hamilton, Marion, and Sequatchie). Only Tennessee counties were included for the Memphis and Chattanooga MSAs. These MSAs were chosen as they have the highest percentage of African Americans in the state. Only respondents in the Nashville MSA were exposed to a social marketing campaign designed to build COVID-19 vaccine confidence among African Americans.

**Figure 1:**
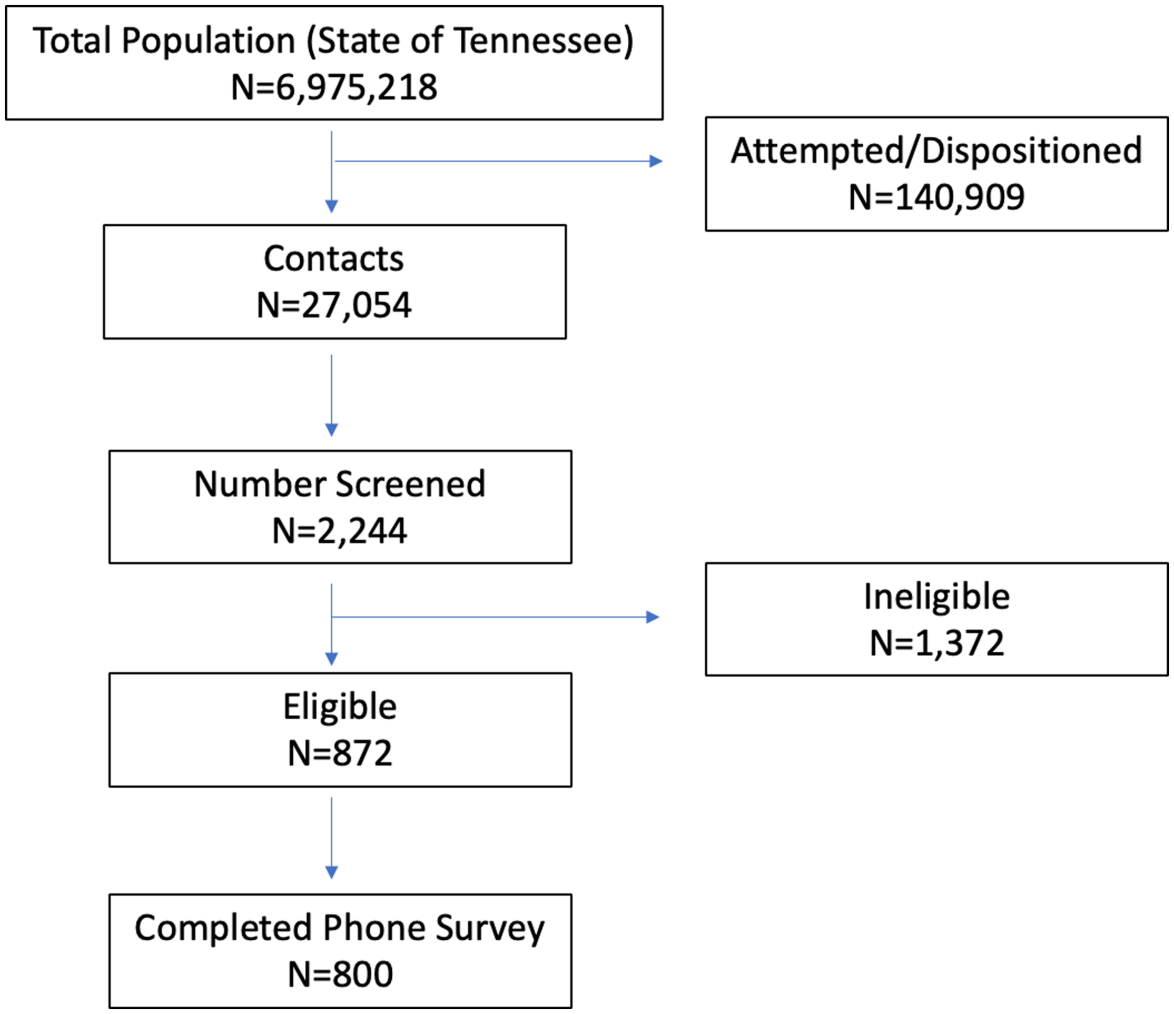
The eligibility screening process for Random Digit Dialing (data source 1).

Data collection was done via random digit dialing, 80% wireless and 20% landline. During the telephone survey, participants were screened for eligibility. If eligible, participants provided verbal consent before completing the telephone survey. Participants received a $20 incentive for their time. See the flow diagram of recruitment processes for data source 1 in Figure 1. The figure for data source 2 (the Nashville MSA and statewide survey of adults with children) is in Supplementary File A.

### 2.3 Measures

Each of the surveys consisted of items and instruments developed by the research team based on prior research (27, 28) and experience and also based on published available items and surveys. In some instances, items were adapted for the study to focus on selected vaccinations and the population being studied. Questions were related to overall vaccine hesitancy and hesitancy specific to COVID-19, influenza, and HPV vaccines. In this study, we present measures for overall vaccine hesitancy, as well as COVID-19 and influenza vaccine hesitancy. There were 9 items related to HPV vaccine hesitancy, and two items will be described in another study. All items included response options “unsure/do not know” or “refused,” and not read by the telephone survey administrator. These two response options were coded but treated as missing in the analysis. The secondary data analysis for this study will not differentiate between the two data sources hereafter but focus on the variables of interest that were available in both datasets.

#### 2.3.1 Vaccine-related predictors

This section provides variables used to identify predictors of influenza and COVID-19 vaccination, including overall vaccine effectiveness, overall vaccine trust, overall vaccine hesitancy (6, 23, 29, 30), preferred sources of vaccine information, preferred place to get vaccines, know someone who died or extremely ill from COVID-19 vaccine, concerned about getting COVID-19, trust in public health agencies that recommended COVID-19 vaccines, influenza vaccine effectiveness, influenza vaccine need, influenza vaccine importance, influenza vaccine safety, race, age, gender, education, income, employment status, health insurance, relationship status, living arrangement, religion, and receipt of influenza vaccine. The categorization of these variables is shown in Supplementary File B.

#### 2.3.2 Outcomes (used in multivariable analyses)

There were two primary outcomes in this study: COVID-19 vaccine receipt and flu vaccine receipt. Each measure is described below. *Receipt of COVID-19 Vaccine:* Respondents were asked, “Have you received a COVID-19 vaccine? [At least one dose of a COVID-19 vaccine. Examples include brand names Pfizer/BioNTech or Comirnaty, Moderna, or Johnson & Johnson.]” (29). Telephone survey administrators could provide additional information about this question, if prompted: “At least one dose of a COVID-19 vaccine. Examples include brand names Pfizer/BioNTech or Comirnaty, Moderna, or Johnson & Johnson.” These were the options for COVID-19 vaccines at the time of the study. Response options were “yes” and “no.”

### 2.4 Data analyses

Descriptive and inferential statistics were performed to analyze study sample data using SAS 9.4 (SAS Institute Inc., Cary, NC). Chi-square tests were used to univariately compare general participant characteristics and vaccine-related factors for two outcome variables (COVID-19 vaccine receipt and influenza vaccine receipt) (Tables 1, 2). Two multivariable logistic regression models were used to estimate adjusted odds ratios (aORs) and 95% confidence intervals (CIs) to predict sociodemographic and overall vaccine-related factors associated with receipt or non-receipt (referent) of the COVID-19 and influenza vaccines. Our rationale for utilizing logistic regression was to obtain odds ratios for the dichotomous outcome variables that could be explained by potential predictors. Assumptions of logistic regression met in the study include the independence of errors, absence of multicollinearity, and lack of strongly influential outliers. Multivariable models of the association between potential predictors and COVID-19 and influenza vaccine status were adjusted for age, gender, race/ethnicity, annual household income, and source as a proxy for survey date completion (Tables 3, 4). value of ps for trend were calculated for ordered categorical variables by examining the odds ratios moving from the lowest referent group to the next highest category and so on for a minimum of three categories. A significance level of alpha = 0.05 was selected. An example of our multivariable model to determine the association between categories of education and COVID vaccination while adjusting for age, gender, race/ethnicity, annual household income, and source in Table 3 is as follows:


$$\begin{array}{l} \mathrm{COVID vaccination}=\alpha +\beta 1\cdot \mathrm{education}\;\left(\mathrm{some college}\right)\\ \;+\beta 2\cdot \mathrm{education}\;\left(\mathrm{college or above}\right)+\beta 3\cdot \mathrm{age}\;\left(35-49\right)\\ \;+\beta 4\cdot \mathrm{age}\;\left(50-65\right)+\beta 5\cdot \mathrm{gender}\;\left(\mathrm{men}\right)+\beta 6\cdot \mathrm{race}/\mathrm{ethnicity}\\ \;\left(\mathrm{black}\right)+\beta 7\cdot \mathrm{race}/\mathrm{ethnicity}\;\left(\mathrm{all}\;\mathrm{other groups}\right)+\beta 8\cdot \mathrm{annual}\\ \;\mathrm{household income}\;\left(\$40,000-\$79,999\right)+\beta 9\cdot \mathrm{annual}\\ \;\mathrm{household income}\;\left(\$80.000\right)+\beta 10\cdot \mathrm{annual household}\\ \;\mathrm{income}\;\left(\mathrm{refused}\right)+\beta 11\cdot \mathrm{source}\;\left(\mathrm{parent Nashville survey}\right)\\ \;+\beta 12\cdot \mathrm{source}\;\left(\mathrm{parent Statewide survey}\right)\text{.} \end{array}$$

**Table 1:** Characteristics of study participants and COVID-19 vaccine receipt.

	No, *n* = 309 *n* (%)	Yes, reported COVID-19 vaccine *n* = 1,088 *n* (%)	*p*-value
Age			0.0007^***^
18–34	48 (15.5)	106 (9.7)	
35–49	191 (61.8)	636 (58.5)	
50–65	70 (22.7)	346 (31.8)	
Gender			0.25
Women	189 (62.8)	719 (66.3)	
Men	112 (37.2)	365 (33.7)	
Race/ethnicity			0.0003^***^
White	206 (68.4)	671 (62.1)	
Black	68 (22.6)	355 (32.9)	
All other groups	27 (9.0)	54 (5.0)	
Annual household income			<0.0001^****^
<$40,000	78 (25.7)	169 (15.7)	
$40,000 to $79,999	84 (27.6)	270 (25.1)	
$80,000+	127 (41.8)	585 (54.4)	
Refused	15 (4.9)	52 (4.8)	
Source			0.001^***^
Adult survey	112 (36.3)	487 (44.8)	
Parent Nashville survey	60 (19.4)	240 (22.0)	
Parent statewide survey	137 (44.3)	361 (33.2)	
Education			<0.0001^****^
High school or lower	62 (20.2)	127 (11.7)	
Some college	117 (38.1)	277 (25.5)	
College or above	128 (41.7)	683 (62.8)	
Employment status			0.0002^***^
Full-time	199 (65.0)	809 (74.5)	
Part-time/unemployed	68 (22.2)	140 (12.9)	
Retired, student, disabled	38 (12.8)	137 (12.6)	
Health insurance status			<0.0001^****^
No	31 (10.1)	39 (3.6)	
Yes	275 (89.9)	1,048 (96.4)	
Without health insurance in past year			<0.0001^****^
No	255 (83.1)	938 (91.8)	
Yes	52 (16.9)	89 (8.2)	
Relationship status			0.03^*^
Single	51 (16.7)	192 (17.8)	
Married	194 (63.4)	739 (68.3)	
Divorced, separated, widowed	61 (19.9)	150 (13.9)	
Living arrangement			0.03^*^
Lives alone	20 (6.5)	96 (8.9)	
Lives with spouse only	19 (6.2)	117 (10.8)	
Lives with spouse and child(ren)	177 (57.6)	609 (56.4)	
Lives with child(ren) only	62 (20.2)	164 (15.2)	
Lives with others	29 (9.5)	94 (8.7)	
Importance of religion			0.008^**^
Not at all	29 (9.5)	143 (13.2)	
Slightly	13 (4.3)	87 (8.1)	
Moderately	49 (16.1)	152 (14.1)	
Very	76 (24.9)	305 (28.2)	
Extremely	138 (45.2)	394 (36.4)	
Overall vaccine effectiveness			<0.0001^****^
Not at all	31 (10.2)	4 (0.4)	
A little	42 (13.9)	23 (2.1)	
Somewhat	144 (47.5)	224 (20.7)	
Very	86 (28.4)	833 (76.8)	
Overall vaccine trust			<0.0001^****^
Not at all	63 (20.6)	4 (0.4)	
A little	52 (17.0)	18 (1.6)	
Somewhat	78 (25.5)	129 (11.9)	
Mostly	101 (33.0)	556 (51.3)	
Completely	12 (3.9)	377 (34.8)	
Overall vaccine hesitancy			<0.0001^****^
Not at all	41 (13.4)	666 (61.5)	
A little	77 (25.1)	222 (20.5)	
Somewhat	92 (26.7)	157 (14.5)	
Very	107 (34.8)	38 (3.5)	
Information about vaccines			<0.0001^****^
Primary care providers	197 (65.9)	806 (74.6)	
CDC/State health departments	24 (8.1)	136 (12.6)	
News sources (e.g., television, Internet, and radio) and online publishers of medical information (e.g., WebMD or Mayo Clinic)	34 (11.4)	70 (6.5)	
Other	44 (14.7)	68 (6.3)	
Preferred place to get vaccine			0.0009^***^
Doctor’s office	217 (75.4)	701 (66.3)	
Health Department	17 (5.9)	76 (7.2)	
Hospital	19 (6.6)	48 (4.5)	
Free standing retail pharmacy	9 (3.1)	86 (8.1)	
In-store pharmacy	6 (2.1)	66 (6.2)	
Other (including workplace)	20 (6.9)	81 (7.7)	
Received influenza vaccine this season			<0.0001^****^
No	262 (85.3)	345 (31.9)	
Yes	45 (14.7)	738 (68.1)	
Concerned about getting COVID-19			<0.0001^****^
Not at all	198 (64.5)	334 (30.8)	
A little	36 (11.7)	198 (18.3)	
Somewhat	28 (9.1)	257 (23.7)	
Very	45 (14.7)	295 (27.2)	
Trust in public health agencies that recommended COVID-19 vaccine			<0.0001^****^
Not at all	173 (56.7)	74 (6.8)	
A little	74 (24.3)	117 (10.8)	
Somewhat	48 (15.7)	403 (37.2)	
Very much	10 (3.3)	489 (45.2)	
Know someone who became seriously ill or died of COVID-19			0.001^***^
No	129 (41.9)	349 (32.1)	
Yes	179 (58.1)	739 (67.9)	

^*^p ≤ 0.05; ^**^p ≤ 0.01; ^***^p ≤ 0.001; ^****^p ≤ 0.0001.

**Table 2:** Association between sociodemographic and vaccine-related factors and COVID-19 vaccine receipt.

	No %	Yes, reported COVID-19 vaccine %	aOR (95% CI)*
Age			
18–34	15.5	9.7	1.0 (referent)
35–49	61.8	58.5	1.4 (0.9–2.2)
50–65	22.7	31.8	**1.9 (1.2–3.2)**
*p*-value for trend			0.03^*^
Gender			
Women	62.8	66.3	1.0 (referent)
Men	37.2	33.7	0.9 (0.6–1.2)
Race/ethnicity			
White	68.4	62.1	1.0 (referent)
Black	22.6	32.9	**2.0 (1.3–2.8)**
All other groups	9.0	5.0	0.6 (0.3–1.0)
Annual household income			
<$40,000	25.7	15.7	1.0 (referent)
$40,000 to $79,999	27.6	25.1	**1.6 (1.1–2.4)**
$80,000+	41.8	54.4	**2.7 (1.8–4.0)**
Refused	4.9	4.8	1.8 (0.8–4.1)
*p*-value for trend			<0.0001^****^
Source			
Adult survey	36.3	44.8	1.0 (referent)
Parent Nashville survey	19.4	22.0	0.9 (0.6–1.4)
Parent statewide survey	44.3	33.2	**0.6 (0.4–0.9)**
Education			
High school or lower	20.2	11.7	1.0 (referent)
Some college	38.1	25.5	1.1 (0.7–1.8)
College or above	41.7	62.8	**2.3 (1.5–3.6)**
*p*-value for trend			<0.0001^*^
Employment status			
Full-time	65.0	74.5	1.0 (referent)
Part-time/unemployed	22.2	12.9	**0.5 (0.4–0.8)**
Retired, student, disabled	12.8	12.6	0.9 (0.5–1.5)
Health insurance status			
No	10.1	3.6	1.0 (referent)
Yes	89.9	96.4	**2.2 (1.2–4.1)**
Without health insurance in past year			
No	83.1	91.8	1.0 (referent)
Yes	16.9	8.2	**0.5 (0.3–0.8)**
Relationship status			
Single	16.7	17.8	1.0 (referent)
Married	63.4	68.3	0.9 (0.5–1.4)
Divorced, separated, widowed	19.9	13.9	0.6 (0.3–1.0)
Living arrangement			
Lives alone	6.5	8.9	1.0 (referent)
Lives with spouse only	6.2	10.8	1.3 (0.5–2.9)
Lives with spouse and child(ren)	57.6	56.4	0.7 (0.3–1.4)
Lives with child(ren) only	20.2	15.2	0.6 (0.3–1.2)
Lives with others	9.5	8.7	0.8 (0.4–1.7)
Importance of religion			
Not at all	9.5	13.2	1.0 (referent)
Slightly	4.3	18.1	1.3 (0.6–2.7)
Moderately	16.1	14.1	0.6 (0.3–1.1)
Very	24.9	28.2	0.8 (0.5–1.4)
Extremely	45.2	36.4	**0.5 (0.3–0.9)**
*p*-value for trend			0.008^**^
Overall vaccine effectiveness			
Not at all/A little	24.1	2.5	1.0 (referent)
Somewhat	47.5	20.7	**2.7 (1.5–4.8)**
Very	28.4	76.8	**18.0 (10.1–31.9)**
*p*-value for trend			<0.0001^****^
Overall vaccine trust			
Not at all/A little	37.6	2.0	1.0 (referent)
Somewhat	25.5	11.9	**6.7 (3.7–12.2)**
Mostly	33.0	51.3	**23.6 (13.3–42.0)**
Completely	3.9	34.8	**151.2 (66.2–345.2)**
*p*-value for trend			<0.0001^****^
Overall vaccine hesitancy			
Not at all	13.4	61.5	1.0 (referent)
A little	25.1	20.5	**0.2 (0.1–0.3)**
Somewhat	26.7	14.5	**0.1 (0.07–0.2)**
Very	34.8	3.5	**0.02 (0.01–0.04)**
*p*-value for trend			<0.0001^****^
Information about vaccines			
Primary care providers	65.9	74.6	1.0 (referent)
CDC/State health departments	8.0	12.6	1.3 (0.8–2.3)
News sources (e.g., television, Internet, and radio) and online publishers of medical information (e.g., WebMD or Mayo Clinic)	11.4	6.5	**0.5 (0.3–0.8)**
Other	14.7	6.3	**0.3 (0.2–0.5)**
Preferred place to get vaccine			
Doctor’s office	75.4	66.3	1.0 (referent)
Health Department	5.9	7.2	1.4 (0.8–2.7)
Hospital	6.6	4.5	1.0 (0.5–2.0)
Free standing retail pharmacy	3.1	8.1	**4.2 (1.8–10.0)**
In-store pharmacy	2.1	6.2	**3.1 (1.2–7.9)**
Other (including workplace)	6.9	7.7	1.5 (0.8–2.7)

*Odds ratios adjusted for age, gender, race/ethnicity, annual household income, and source.

Significant odds ratios are bolded. *p ≤ 0.05; **p ≤ 0.01; ***p ≤ 0.001; ****p ≤ 0.0001.

**Table 3:** Characteristics of study participants and influenza vaccine receipt.

	No *n* = 610 *n* (%)	Yes, reported influenza vaccine *n* = 783 *n* (%)	*p*-value
Age			0.0008^***^
18–34	74 (12.1)	77 (9.8)	
35–49	386 (63.3)	441 (56.3)	
50–65	150 (24.6)	265 (33.8)	
Gender			0.11
Women	382 (63.3)	523 (67.3)	
Men	222 (36.7)	254 (32.7)	
Race/ethnicity			0.57
White	375 (62.7)	503 (64.7)	
Black	185 (30.9)	235 (30.2)	
All other groups	38 (6.4)	40 (5.1)	
Annual household income			0.002^**^
<$40,000	124 (20.7)	123 (15.8)	
$40,000 to $79,999	171 (28.6)	180 (23.1)	
$80,000+	275 (45.9)	437 (56.2)	
Refused	29 (4.8)	38 (4.9)	
Source			0.05^*^
Adult survey	242 (39.7)	353 (45.1)	
Parent Nashville survey	128 (21.0)	170 (21.7)	
Parent statewide survey	240 (39.3)	260 (33.2)	
Education			0.001^***^
High school or lower	90 (14.8)	98 (12.5)	
Some college	198 (32.5)	195 (25.0)	
College or above	321 (52.7)	488 (62.5)	
Employment status			0.002^**^
Full-time	429 (70.8)	576 (73.6)	
Part-time/unemployed	112 (18.5)	96 (12.3)	
Retired, student, disabled	65 (10.7)	110 (14.1)	
Health insurance status			<0.0001^****^
No	50 (8.3)	19 (2.4)	
Yes	556 (91.7)	764 (97.6)	
Without health insurance in past year			<0.0001^****^
No	514 (84.5)	736 (94.1)	
Yes	94 (15.5)	46 (5.9)	
Relationship status			0.20
Single	117 (19.3)	124 (15.9)	
Married	403 (66.6)	529 (68.0)	
Divorced, separated, widowed	85 (14.1)	125 (16.1)	
Living arrangement			0.17
Lives alone	42 (6.9)	73 (9.4)	
Lives with spouse only	52 (8.6)	83 (10.7)	
Lives with spouse and child(ren)	346 (57.1)	439 (56.5)	
Lives with child(ren) only	110 (18.2)	116 (14.9)	
Lives with others	56 (9.2)	66 (8.5)	
Importance of religion			0.07
Not at all	69 (11.4)	102 (13.1)	
Slightly	35 (5.8)	64 (8.3)	
Moderately	80 (13.2)	119 (15.3)	
Very	165 (27.3)	215 (27.7)	
Extremely	256 (42.3)	276 (35.6)	
Overall vaccine effectiveness			<0.0001^****^
Not at all	32 (5.3)	3 (0.4)	
A little	47 (7.8)	16 (2.1)	
Somewhat	225 (37.3)	143 (18.3)	
Very	299 (49.6)	618 (79.2)	
Overall vaccine trust			<0.0001^****^
Not at all	61 (10.1)	5 (0.7)	
A little	60 (9.9)	10 (1.3)	
Somewhat	133 (22.0)	76 (9.7)	
Mostly	268 (44.2)	386 (49.4)	
Completely	84 (13.8)	304 (38.9)	
Overall vaccine hesitancy			<0.0001^****^
Not at all	174 (28.8)	529 (67.7)	
A little	160 (26.4)	137 (17.5)	
Somewhat	149 (24.6)	91 (11.7)	
Very	122 (20.2)	24 (3.1)	
Information about vaccines			<0.0001^****^
Primary care providers	411 (68.7)	588 (75.8)	
CDC/State health departments	57 (9.5)	103 (13.3)	
News sources (e.g., television, Internet, and radio) and online publishers of medical information (e.g., WebMD or Mayo Clinic)	61 (10.2)	42 (5.4)	
Other	69 (11.6)	43 (5.5)	
Preferred place to get vaccine			0.09
Doctor’s office	404 (69.4)	510 (67.1)	
Health Department	42 (7.2)	51 (6.7)	
Hospital	34 (5.8)	33 (4.4)	
Free standing retail pharmacy	28 (4.8)	67 (8.8)	
In-store pharmacy	30 (5.2)	42 (5.5)	
Other (including workplace)	44 (7.6)	57 (7.5)	
Received COVID vaccine			<0.0001^****^
No	262 (43.2)	45 (5.8)	
Yes	345 (56.8)	738 (94.2)	
Influenza vaccine effectiveness			<0.0001^****^
Not at all	114 (19.9)	9 (1.2)	
A little	151 (26.3)	32 (4.1)	
Somewhat	223 (38.8)	354 (45.8)	
Very	86 (15.0)	378 (48.9)	
Influenza vaccine need			<0.0001^****^
Not at all	182 (30.3)	17 (2.2)	
A little	139 (23.1)	40 (5.2)	
Somewhat	212 (35.3)	259 (33.4)	
Very	68 (11.3)	459 (59.2)	
Influenza vaccine importance			<0.0001^****^
Not at all	164 (27.1)	15 (1.9)	
A little	132 (21.8)	33 (4.2)	
Somewhat	219 (36.2)	213 (27.3)	
Very	90 (14.9)	520 (66.6)	
Influenza vaccine safety			<0.0001^****^
Not at all	68 (11.6)	3 (0.4)	
A little	72 (12.3)	12 (1.5)	
Somewhat	219 (37.3)	104 (13.4)	
Very	228 (38.8)	660 (84.7)	

*p ≤ 0.05; **p ≤ 0.01; ***p ≤ 0.001; ****p ≤ 0.0001.

**Table 4:** Association between sociodemographic and vaccine-related factors and influenza vaccine receipt.

	No %	Yes, reported influenza vaccine %	aOR (95% CI)*
Age			
18–34	12.1	9.8	1.0 (referent)
35–49	63.3	56.3	1.0 (0.7–1.4)
50–65	24.6	33.8	1.5 (1.0–2.3)
*p*-value for trend			0.007^**^
Gender			
Women	63.3	67.3	1.0 (referent)
Men	36.7	32.7	1.0 (0.7–1.0)
Race/ethnicity			
White	62.7	64.7	1.0 (referent)
Black	30.9	30.2	1.0 (0.7–1.3)
All other groups	6.4	5.1	0.9 (0.5–1.4)
Annual household income			
<$40,000	20.7	15.8	1.0 (referent)
$40,000 to $79,999	28.6	23.1	1.2 (0.8–1.7)
$80,000+	45.9	56.2	**1.9 (1.3–2.6)**
Refused	4.8	4.9	1.3 (0.7–2.5)
*p*-value for trend			0.0006^***^
Source			
Adult survey	39.7	45.1	1.0 (referent)
Parent Nashville survey	21.0	21.7	0.9 (0.6–1.2)
Parent statewide survey	39.3	33.2	0.8 (0.6–1.1)
Education			
High school or lower	14.8	12.5	1.0 (referent)
Some college	32.5	25.0	0.8 (0.5–1.2)
College or above	52.7	62.5	1.2 (0.8–1.8)
*p*-value for trend			0.02^*^
Employment status			
Full-time	70.8	73.6	1.0 (referent)
Part-time/unemployed	18.5	12.3	**0.7 (0.5–0.9)**
Retired, student, disabled	10.7	14.1	1.3 (0.9–1.9)
Health insurance status			
No	8.3	2.4	1.0 (referent)
Yes	91.7	97.6	**2.6 (1.4–4.8)**
Without health insurance in the past year			
No	84.5	94.1	1.0 (referent)
Yes	15.5	5.9	**0.4 (0.3–0.7)**
Relationship status			
Single	19.3	15.9	1.0 (referent)
Married	66.6	68.0	1.0 (0.7–1.4)
Divorced, separated, widowed	14.1	16.1	1.3 (0.8–2.0)
Living arrangement			
Lives alone	6.9	9.4	1.0 (referent)
Lives with spouse only	8.6	10.7	0.7 (0.4–1.2)
Lives with spouse and child(ren)	57.1	56.5	0.6 (0.4–1.1)
Lives with child(ren)	18.2	14.9	0.7 (0.4–1.2)
Lives with others	9.2	8.5	0.8 (0.4–1.3)
Importance of religion			
Not at all	11.4	13.1	1.0 (referent)
Slightly	5.8	8.3	1.2 (0.7–2.1)
Moderately	13.2	15.3	1.0 (0.6–1.5)
Very	27.3	27.7	0.8 (0.6–1.3)
Extremely	42.3	35.6	0.7 (0.5–1.0)
*p*-value for trend			0.08
Overall vaccine effectiveness			
Not at all/A little	13.1	2.5	1.0 (referent)
Somewhat	37.3	18.3	**2.6 (1.4–5.1)**
Very	49.6	79.2	**8.1 (4.3–15.1)**
*p*-value for trend			<0.0001^****^
Overall vaccine trust			
Not at all/A little	20.0	2.0	1.0 (referent)
Somewhat	22.0	9.7	**4.7 (2.3–9.7)**
Mostly	44.2	49.4	**12.3 (6.2–24.4)**
Completely	13.8	38.9	**30.8 (15.1–62.7)**
*p*-value for trend			<0.0001^****^
Overall vaccine hesitancy			
Not at all	28.8	67.7	1.0 (referent)
A little	26.4	17.5	**0.3 (0.2–0.4)**
Somewhat	24.6	11.7	**0.2 (0.1–0.3)**
Very	20.2	3.1	**0.07 (0.04–0.1)**
*p*-value for trend			<0.0001^****^
Information about vaccines			
Primary care providers	68.7	75.8	1.0 (referent)
CDC/State health departments	9.5	13.3	1.2 (0.8–1.8)
News sources (e.g., television, Internet, and radio) and online publishers of medical information (e.g., WebMD or Mayo Clinic)	10.2	5.4	**0.5 (0.3–0.8)**
Other	11.6	5.5	**0.4 (0.3–0.6)**
Preferred place to get vaccine			
Doctor’s office	69.4	67.1	1.0 (referent)
Health Department	7.2	6.7	1.1 (0.7–1.8)
Hospital	5.8	4.4	0.9 (0.5–1.6)
Free standing retail pharmacy	4.8	8.8	**1.8 (1.1–2.9)**
In-store pharmacy	5.2	5.5	1.2 (0.7–2.0)
Other (including workplace)	7.6	7.5	1.1 (0.7–1.7)

*Odds ratios adjusted for age, gender, race/ethnicity, annual household income, and source.

Significant odds ratios are bolded. *p ≤ 0.05; **p ≤ 0.01; ***p ≤ 0.001; ****p ≤ 0.0001.

The associations for the above model are presented as odds ratios for the exposure of education, comparing the referent group of high school or lower to the comparison groups of some college and college or above.

*p*-values for trend were calculated for ordered categorical variables by examining the odds ratios moving from the lowest referent group to the next highest category and so on for a minimum of three categories. To assess the trend of increasing COVID-19 vaccination with increasing categories of education while adjusting for age, gender, race/ethnicity, annual household income, and source, all variables were treated continuously rather than categorically. An example of our multivariable model testing for the trend is:


$$\begin{array}{l} \mathrm{COVID}\;\mathrm{vaccination}=\alpha +\beta 1\cdot \mathrm{education}+\beta 2\cdot \mathrm{age}\\ \;+\beta 3\cdot \mathrm{gender}+\beta 4\cdot \mathrm{race}/\mathrm{ethnicity}+\beta 5\cdot \mathrm{annual}\\ \;\mathrm{household}\;\mathrm{income}+\beta 6\cdot \mathrm{source}\text{.} \end{array}$$

The test for trend is based on the *p*-value from the Wald test for the trend for the exposure of education modeled continuously rather than as an ordered categorical variable. A significance level of alpha = 0.05 was selected.

## 3 Results

### 3.1 Characteristics of study participants and COVID-19 vaccine receipt

Approximately 78% of the adult sample had received at least one dose of the COVID-19 vaccine. Additional characteristics of COVID-19 vaccine receipt are detailed in Table 1. The proportion of participants who had received the COVID-19 vaccine significantly differed by all characteristics except gender. For example, COVID-19 vaccine receipt was significantly associated with older age (*p* = 0.0007), black race/ethnicity (*p* = 0.0003), and higher annual household income (*p* < 0.0001).

### 3.2 Association between sociodemographic and vaccine-related factors and COVID-19 vaccine receipt

After adjusting for age, gender, race/ethnicity, and annual household income and source, relationship status and living arrangement no longer differed significantly by COVID-19 receipt (Table 2). However, there were differences in sociodemographics and vaccine-related factors. For example, compared to adults aged 18–34 years, adults aged 50–65 years were more likely to receive the COVID-19 vaccine (aOR = 1.9; 95% CI: 1.2–2.3) and there was a significant positive trend for the likelihood of COVID-19 vaccination receipt across age groups (*p* = 0.03). Blacks were twice (95% CI: 1.3–2.8) as likely to receive the COVID-19 vaccine as whites. As annual household income increased, there was a significant positive trend (<0.0001) of vaccination with participants who made $40,000 to $79,999 and $80,000+, being almost two (aOR = 1.6; 95% CI: 1.1–2.4) and three (aOR = 2.7; 95% CI: 1.8–4.0) times more likely to receive the vaccine compared to those who made <$40,000. Similarly, trends of COVID-19 vaccination increased as education increased (*p* < 0.0001), with rates being significantly higher among those who had a college degree or above (aOR = 2.3; 95% CI: 1.5–3.6) compared to those who had a high school degree or lower. Those who were extremely religious were significantly less likely to be vaccinated (aOR = 0.5; 95% CI: 0.3–0.9) compared to individuals who were not at all religious, and there was a significant trend of decreasing likelihood of COVID-19 receipt with increasing religiosity (*p* = 0.008). As it relates to the source of the survey, adults from the statewide parent survey were less likely to be vaccinated (aOR = 0.6; 95% CI: 0.4–0.9) compared to those on the adult survey.

All vaccine-related factors differed significantly by COVID-19 vaccination receipt after adjustment. For example, relative to persons who indicated they believed the overall vaccine effectiveness and their overall vaccine trust were not at all/a little, there were strongly significant positive trends for the likelihood of COVID-19 receipt with increasing belief in effectiveness (*p* < 0.0001) and trust (*p* < 0.0001). The opposite was true for overall vaccine hesitancy (*p* < 0.0001) (see Table 2).

### 3.3 Characteristics of study participants and influenza vaccine receipt

Over 56% of the adult sample had received the influenza vaccination this season. Additional characteristics of influenza vaccine receipt are detailed in Table 3. The proportion of participants who had received the influenza vaccine differed significantly by most characteristics, excluding gender, race/ethnicity, source, relationship status, living arrangement, importance of religion, and preferred place to get the vaccine. For example, influenza vaccine receipt was significantly associated with older age (*p* = 0.0008), higher annual household income (*p* = 0.002), and having a college degree or above (*p* = 0.001).

### 3.4 Association between sociodemographic and vaccine-related factors and influenza vaccine receipt

After adjusting for age, gender, race/ethnicity, annual household income and source, age, and education no longer differed significantly by influenza vaccine receipt (Table 4). Significantly fewer adults who had received the influenza vaccine were employed part-time or unemployed (aOR = 0.7; 95% CI: 0.5–0.9) compared to those employed full-time. Compared to an income less than $40,000, influenza vaccination was significantly higher among adults whose income was $80,000 or more (aOR = 1.9; 95% CI: 1.3–2.6) and demonstrated a strongly significant positive trend for the likelihood of COVID-19 receipt with increasing income (*p* = 0.0006). Persons who reported having health insurance were 2.6 (95% CI: 1.4–4.8) times more likely to be vaccinated for influenza, while those reporting not having insurance in the past year were 0.4 (95% CI: 0.3–0.7) times less likely to be vaccinated against influenza. All vaccine-related factors differed significantly by influenza receipt after adjustment. Relative to persons who indicated they believed the overall vaccine effectiveness and their overall vaccine trust was not at all/a little, there were strongly significant positive trends for the likelihood of influenza receipt with increasing belief in effectiveness (*p* < 0.0001) and trust (*p* < 0.0001). The opposite was true for overall vaccine hesitancy (*p* < 0.0001). As it relates to information sources for vaccines, those whose information source was news sources and online publishers of medical information (aOR = 0.5; 95% CI: 0.3–0.8) along with other information sources (aOR = 0.4; 95% CI: 0.3–0.6) were significantly less likely to be vaccinated compared to individuals selecting primary care providers. Finally, individuals whose preferred place to get vaccinated was at a free-standing retail pharmacy were significantly more likely to get the vaccine (aOR = 1.8; 95% CI: 1.1–2.9) compared to those who selected the doctor’s office.

## 4 Discussion

The aim of this study was to explore factors related to influenza and COVID-19 vaccine uptake among adults and parents in Tennessee. This random digit dial study is the first to explore predictors of vaccination by type and, if they differ, in the state. Similar to past studies (31), we found that influenza vaccination rates were significantly lower than COVID-19 rates, with nearly 56 and 88% of respondents reporting they received the influenza and COVID-19 vaccines, respectively. While COVID-19 vaccination rates were high among these participants, influenza vaccine rates reflected the long-standing rates at approximately 50%. This does not reflect the potential increase in influenza vaccination and suboptimal COVID-19 vaccination rates during the COVID-19 pandemic as identified in past studies (32, 33), which suggests pockets of disparities in uptake. A few US studies have found geographic location, doctor recommendation, the push to get the vaccine during the COVID-19 pandemic, Internet access, and geographic location to be associated with higher rates of COVID-19 vaccination compared to influenza vaccination rates (31, 34). Overall, this finding warrants more exploration.

Attitudes and beliefs continue to impact COVID-19 vaccine uptake. In our study, overall trust in vaccines, hesitancy related to vaccines in general, and concern about getting COVID-19 were associated with receipt. Of interest, there were multiple reasons for hesitancy, trust in vaccination, and concern about COVID-19 associated with vaccine receipt. Our findings suggest that individuals may not need complete trust or high levels of perceived effectiveness in the vaccine to get vaccinated. However, any degree of hesitancy can negatively impact one’s intent to get vaccinated. Therefore, efforts (i.e., education, community engagement, and patient-provider communication) should continue to build trust in vaccines in general, increase the perceived effectiveness of the vaccine, and address factors influencing hesitancy for increasing uptake.

Similar to a past study in Tennessee (35), sociodemographics were associated with COVID-19 vaccine receipt. Unsurprisingly, being older and Black were more likely to be vaccinated. It has long been studied that older people understand the potential impact of COVID-19 on their health, their immunity to misinformation, and their increased access to Medicare and their early eligibility. In addition, the historical context of research abuse and mistreatment in healthcare negatively impacts the engagement of Black Americans in research preventive behaviors and research. Consistent with past studies (36), we also found that COVID-19 vaccine receipt was significantly higher among those with higher education levels. This may reflect having greater access to information on COVID-19 and the vaccine, along with the ability to identify information sources and trusted messengers (37). It was somewhat surprising that participants who had higher incomes, health insurance, health insurance in the past year specifically, and part-time employment were more likely to be vaccinated; however, this could be due to lack of geographical access to the vaccine since it was free during this time period or the aforementioned confidence issues in the vaccine. However, there was no significant relationship between COVID-19 receipt and gender. This finding suggests a shift in gender-specific barriers to COVID-19 vaccination, which may lead to improved attitudes related to uptake.

To date, there is limited information on psychological factors influencing influenza vaccination receipt among Tennesseans. In our study, we found that individuals who were very hesitant about vaccines in general were less likely to be vaccinated. These findings confirm that negative attitudes toward vaccination, such as influenza vaccination, are significant barriers to uptake (38, 39). However, continuing to increase overall perceived effectiveness and trust in vaccines can positively influence the uptake of all vaccines and influenza vaccines specifically similar to COVID-19 vaccination among our participants. Bhugra et al. identified strategies to improve influenza vaccination coverage that exist at the patient, provider, practice, and policy levels. These strategies include education at the patient, provider, and practice levels and mandates at the policy level. Multi-level strategies will be necessary to optimize receipt (40).

Influenza vaccination receipt was associated with sociodemographic characteristics, namely age, income, and education. In the adjusted model, influenza vaccination was more likely to be among individuals who were older, had a high income, and had high levels of education. Collectively, the positive association of having a high income and high levels of education suggests that having access to care or trust in healthcare providers is necessary for uptake (41, 42). A surprising finding was that the association between gender, race, and influenza vaccination uptake was not significant. Past studies have demonstrated female participants being less likely to get influenza vaccination, which could potentially be due to experiencing more adverse events (43). In addition, influenza vaccination has been found to be lower consistently among Black Americans in past studies (44). Collectively, these findings warrant further study. Finally, individuals who are employed part-time or unemployed, being significantly less likely to get the influenza vaccine, could very well suggest that employers working full-time are receiving education and being offered the influenza vaccines through clinics at work at higher rates.

### 4.1 Strengths and limitations

The strengths of this study include using a random digit dial project to gather a representative sample of the largest MSAs in Tennessee. However, there were some limitations, including limiting the survey to three MSAs, accounting for 20 of the state’s 95 counties, and the findings may not be representative of the general population. This study is limited by self-reported data, which can be influenced by social desirability and/or recall bias. Additionally, this study is limited by cross-sectional data that cannot be used to infer causality and may not be generalizable to other populations. Finally, we collected attitudes about COVID-19 vaccination from those unvaccinated only, a small number, due to the limitations of random digit dial. This limited our ability to conduct analyses on this data. Therefore, future studies should assess whether attitudes differ toward COVID-19 and influenza vaccination among adults in Tennessee to further inform intervention strategies.

## 5 Conclusion

This is one of the first studies to explore factors influencing uptake by vaccine type at the state level. Understanding factors influencing uptake by vaccines is integral to developing strategies that can be applied across vaccines and to each vaccine specifically. The results suggest that strategies to increase COVID-19 and influenza vaccinations should be age-specific, centered around increasing geographical and financial access, and offer tailored messages to address concerns for vaccines overall. However, the newness of the COVID-19 vaccine, along with the historical mistreatment of Black Americans in research and healthcare, might require multi-level approaches to initiate equity in these systems. These actions can positively impact perceptions of experiences within these entities and ultimately health outcomes.

## Data availability statement

The datasets presented in this article are not readily available due to confidentiality agreements; therefore, supporting data cannot be made openly available. Requests to access the data should be directed to JC-E, jennifer.erves@vumc.org.

## Ethics statement

The studies involving humans were approved by Meharry Medical College Institutional Review Board. The studies were conducted in accordance with the local legislation and institutional requirements. Written informed consent for participation was not required from the participants or the participants’ legal guardians/next of kin because we had verbal informed consent since data was collected via a Random Digit Dial.

## Author contributions

JC-E: Conceptualization, Funding acquisition, Investigation, Project administration, Resources, supervision, Validation, Writing-original draft, Writing-review and editing, Methodology. WG: Visualization, Writing – original draft, Writing – review & editing. MS: Conceptualization, Formal analysis, Investigation, Methodology, Supervision, Writing – original draft, Writing – review & editing, Data curation, Funding acquisition, Resources, Software, Validation. ES: Visualization, Writing – review & editing. SJ: Funding acquisition, Writing – review & editing, Conceptualization, Investigation, Resources. JD: Conceptualization, Funding acquisition, Investigation, Resources, Supervision, Writing – review & editing. HB: Conceptualization, Funding acquisition, Investigation, Project administration, Resources, Supervision, Validation, Writing – original draft, Writing – review & editing, Methodology.
