# Supplementary material for: Predictors of seasonal influenza and COVID-19 vaccination coverage among adults in Tennessee during the COVID-19 pandemic
Source: Front Public Health. 2024 Mar 4;12:1321173. doi: 10.3389/fpubh.2024.1321173 (PMC10945017; doi:10.3389/fpubh.2024.1321173)
Supplement: Supplementary file 1 [file Data_Sheet_1.docx]

**Supplementary Material**

Supplemental File A:

Figure 2: The eligibility screening process for Random Digit Dialling (Data Source 2)


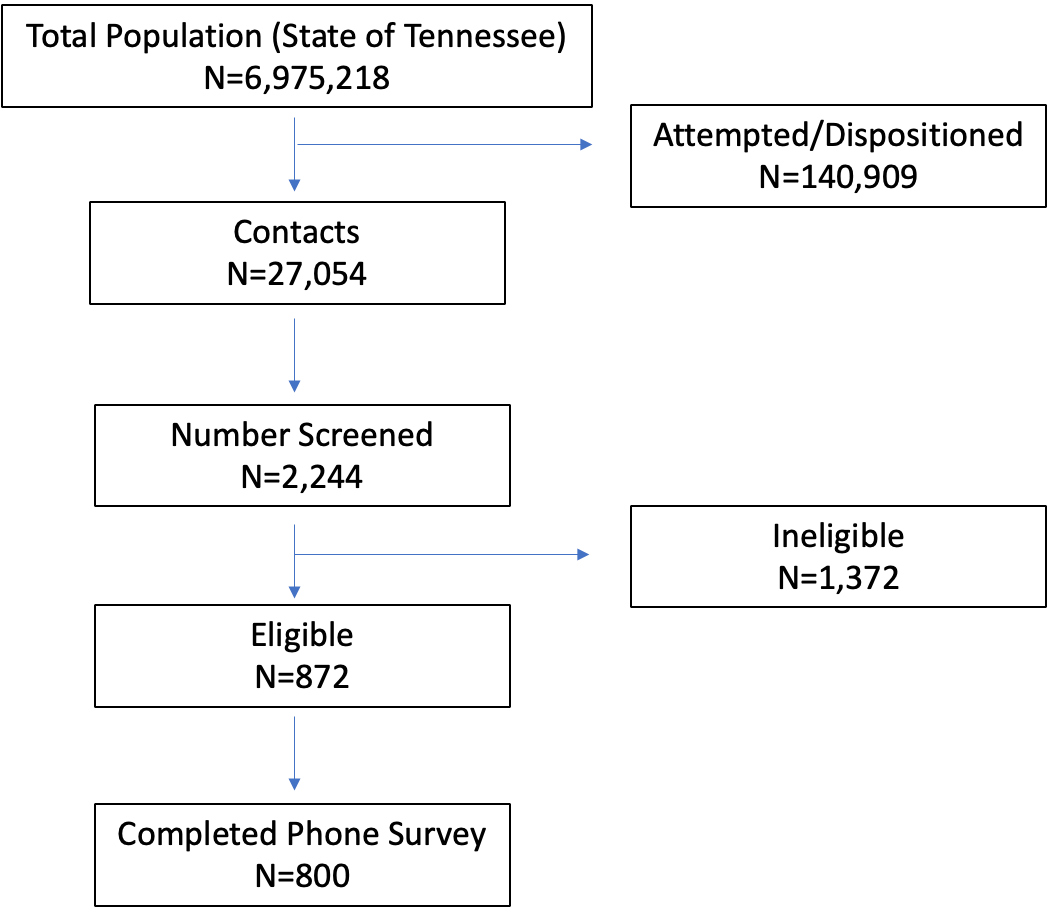


***Supplemental File B:***

***Categorization of predictor variables***

***Overall Perceptions (used in univariate and multivariable analyses):*** The following three items assessed overall vaccine effectiveness, overall vaccine trust, and overall vaccine hesitancy and were adapted from previously published research on vaccination hesitancy.^23, 28-30^ ***Overall Vaccine Effectiveness.*** One item assessed respondents’ perceptions of overall, general vaccine effectiveness. Response options were a 4-point Likert-type scale ranging from “very effective,” “somewhat effective,” “a little effective,” and “not at all effective.” “A little effective” and “not at all effective” were merged for the multivariable analyses. ***Overall Vaccine Trust.*** One item assessed respondents’ overall, general trust in vaccines. Response options were a 5-point Likert scale from “completely,” “mostly,” “somewhat,” “a little,” and “not at all.” “A little” and “not at all” were merged for the multivariable analyses. ***Overall Vaccine Hesitancy.*** One item assessed respondents’ overall, general hesitancy toward vaccines. Response options were a 4-point Likert-type scale ranging from “very hesitant,” “somewhat hesitant,” “a little hesitant,” and “not at all hesitant.”

***Preferred sources of vaccine information (used in univariate and multivariable analyses).*** One item developed by the research team assessed from whom and/or from where respondents preferred to gather information on vaccines in general if they were going to do so. Eighteen response options were provided including 17 potential, predefined, and possible sources of information, such as government organizations, family and friends, online, and “other [please specify]” as a final option. Up to three responses were coded per participant noting first, second, and third responses; however, prioritization of responses was not prompted. For multivariable analyses, first responses were merged into four categories: 1) primary care providers, 2) CDC and state health departments, 3) news sources (e.g., television) and online publishers of medical information (e.g., WebMD), and 4) other (employer, family and friends, FDA, health insurers, hospital system websites, local health officials, nurses, pharmacists, professional organizations, religious leaders, social media, union leaders and other).

***Preferred place to get vaccines (used in univariate and multivariable analyses).*** One item developed by the research team assessed respondents’ preferred place to get vaccines, in general. “If you have a choice, at what kind of place would you prefer to get a vaccine?” was asked. Response options included six predefined locations, such as workplace, doctor’s office, health department clinic, hospital, free standing retail pharmacy, in-store pharmacy, and “other [please specify]” as a final option. For multivariable analyses, workplace was merged with other.

***COVID-19-specific items (used in univariate analyses):*** The following three items assessed specific predictors related to COVID-19 disease and COVID-19 vaccination. Items were adapted from the CDC COVID-19 rapid community assessment tool.^30^ ***Know someone who died or severely ill of COVID-19.*** Respondents reported “yes” or “no” to if they personally knew anyone in your family, group of friends, or community networks who became seriously ill or died as a result of COVID-19. ***Concerned about getting COVID-19.*** One item was used to assess respondents' concern about getting COVID-19. Response options were a 4-point Likert-type scale ranging from “very concerned,” “somewhat concerned,” “a little concerned,” to “not at all concerned.” ***Trust in public health agencies that recommended COVID-19 vaccines.*** One item assessed respondents’ level of trust in public health agencies that recommended COVID-19 vaccination. Response options were a 4-point Likert-type scale ranging from “very much,” “somewhat,” “a little,” to “not at all.”

***Influenza vaccine-specific items (used in univariate analyses):***  The following four items assessed specific beliefs about influenza vaccines. The term flu was used to represent the common language of this vaccine. These four items were adapted from previously published items.^28^ ***Influenza vaccine effectiveness.*** The first item assessed how effective participants think the Influenza vaccine is. Response options were a 4-point Likert scale from ranging from “very effective," “somewhat effective,” “a little effective,” to “not at all effective.” ***Influenza vaccine need.*** One item assessed how necessary the influenza vaccine is. Response options were a 4-point Likert scale from ranging from “very necessary,” “somewhat necessary,” “a little necessary,” to “not at all necessary.” ***Influenza vaccine importance.*** One item assessed how important participants think the flu vaccine is. Response options were a 4-point Likert scale ranging from “very important,” “somewhat important,” “a little important,” to “not at all important.” ***Influenza vaccine safety.*** One item assessed respondents’ beliefs about the safety of flu vaccines. Response options were a 4-point Likert scale from ranging from “very safe,” “somewhat safe,” “a little safe,” to “not at all safe.”

**Sociodemographic predictors (used in univariate and multivariable analyses).** Descriptive characteristic items to describe respondents were developed by the research team. We describe each socio-demographic predictor and their response options below.

***Race***. Participants were asked to describe their race with response options of White, Black or African American, American Indian or Alaska Native, Native Hawaiian, Asian, and some other race with an open-ended option to specify. Race was represented with categories of Black, White, and all other groups.

***Age.*** Participants were asked their age with the statement “Are you between the ages of…?”. “Age was represented with the categories 18-34, 35-49, and 50-65.

***Gender.*** “With which gender do you most identify?” was the question for gender. Male, female, transgender female, transgender male, and gender variant/non-conforming were the categories representing gender. Male and female were the categories representing gender for this analysis; all other categories were treated as missing.

***Education.*** For education, participants were asked “What is the highest grade of school you completed?” with the response options of some high school, GED or high school diploma, associate degree, some college, bachelor’s degree, master’s degree, doctoral/professional degree, unsure/don’t know, and refused. Categories were merged to form the high school or lower, some college, and college or above categories.

***Income.*** The question related to annual household income prior to taxes being taken out had response options of less than $20,000, $20,001 to $40,000, $40,001 to $60,000, $60,001 to $80,000, $80,001 to $100,000, and over $100,000. Categories were merged to be less than $40,000, $40,000 to $79,999, $80,000+, and refused.

***Employment status.*** The question related to current employment status had the response options employed full time, employed part time, unemployed looking for work, unemployed not look for work, retired, student disabled, unsure/don’t know, and refused. Retired, student, and disabled participants were merged due to the low number of participants in these categories. Similarly, part-time and unemployed categories were merged. The final categories were full-time, part-time/unemployed, and retired/student/disabled.

***Health insurance.*** Health insurance status was represented with the answer choices of yes and no. Without health insurance in the past year answer choices were represented with yes and no answer choices.

***Relationship status.*** For relationship status, response options were single or never married, married or living as married, divorced, separated, widowed, other with the option to specify, unsure/don’t know, and refused. Divorced, separated, and widowed were merged to form a category in addition to the single and married categories.

***Living arrangement.*** Response options were “lives alone”, “lives with spouse only”, “lives with spouse and child(ren)”, “lives with child(ren) only," “lives with others”, “unsure/don’t know”, and “refused”.

***Religion.*** A 5-point scale ranging from “not at all” to “extremely” was used to measure how important religion was to the participant.

***Receipt of Influenza Vaccine.*** Respondents were asked, “Did you get a flu vaccine yet this season?” Responses were recorded and specific response options were not read by the telephone survey administrator unless prompted. Response options were “yes,” “intend to get one but have not yet been vaccinated,” and “no.” Due to the timing of surveys outside the influenza vaccine season, persons who indicated they intended to get the influenza vaccine this season were considered to have received the vaccine.
